# Decoding the brain’s ATG8 paralog code: LC3–GABARAP specialization at synapses and the astrocyte–neuron interface

**DOI:** 10.3389/fcell.2026.1762891

**Published:** 2026-02-02

**Authors:** Haneul Choi, Seung-Min Lee, Jin-A. Lee

**Affiliations:** Department of Biological Sciences and Biotechnology, College of Life Sciences and Nanotechnology, Hannam University, Daejeon, Republic of Korea

**Keywords:** autophagy, brain, GABARAP family protein, LC3 family protein, neurological disorders, synapse

## Abstract

Macroautophagy is essential for the long-term health of neurons and astrocytes in the central nervous system (CNS). The six mammalian ATG8 paralogs (LC3A/B/C and GABARAP/GABARAPL1/L2) exhibit an emerging “ATG8 code”—a division of labor among these proteins that assigns specialized roles in the autophagy pathway to each paralog, enabling fine-tuned proteostasis at synapses and the astrocyte–neuron interface. This review synthesizes how LC3 *versus* GABARAP mediate distinct steps of autophagy (LC3 primarily governs cargo recruitment and phagophore expansion, whereas GABARAP drives autophagosome maturation, transport, and lysosomal fusion) and how these molecular distinctions translate into functional differences in neurons *versus* astrocytes. Neurons coordinate autophagy across long axons and synapses: presynaptic autophagy clears aging synaptic vesicles and organelles, while postsynaptic autophagy modulates receptor turnover and synaptic plasticity. Astrocytes, by contrast, leverage autophagy for metabolic support and clearance of extracellular debris (e.g., amyloid-β plaques), interfacing with neuronal autophagy *via* transcellular mechanisms. Dysregulation of these processes underlies diverse CNS disorders: impaired autophagic flux and aggregate clearance contribute to neurodegenerative diseases (Alzheimer’s and Parkinson’s), whereas selective autophagy deficits at synapses disrupt circuit homeostasis (implicated in epilepsy and autism). Finally, we highlight emerging methodologies—from multi-omics and live imaging to optogenetics and targeted therapeutics—that are illuminating this specialized autophagy network and opening novel avenues for intervention.

## 1 Introduction

**FIGURE 1:**
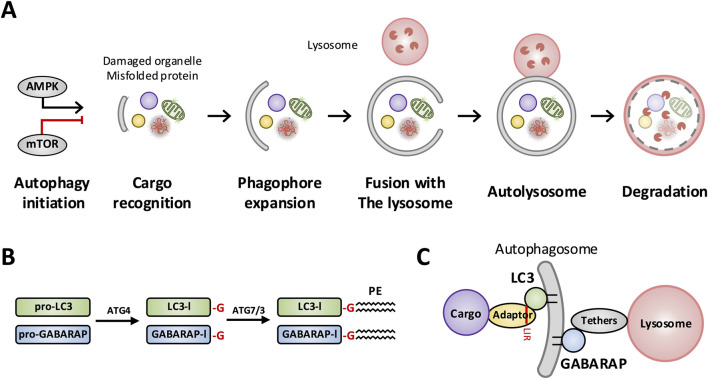
Overview of macroautophagy and ATG8-family (LC3/GABARAP) lipidation during autophagosome maturation and lysosomal fusion. **(A)** Autophagy initiation is regulated by nutrient/energy-sensing kinases, with AMPK promoting and mTOR suppressing autophagy. Upon induction, damaged organelles and misfolded proteins are recognized as cargo and sequestered by the expanding phagophore. The phagophore elongates and closes to form an autophagosome, which subsequently fuses with a lysosome to generate an autolysosome where cargo is degraded. **(B)** Processing and lipid conjugation of ATG8-family proteins. Pro-LC3 and pro-GABARAP are cleaved by ATG4 to expose the C-terminal glycine, producing the cytosolic forms LC3-I and GABARAP-I. Through the ATG7/ATG3 conjugation cascade, LC3 and GABARAP are covalently conjugated to phosphatidylethanolamine (PE), generating membrane-associated LC3-II and GABARAP-II. **(C)** Functional roles of lipidated LC3/GABARAP on autophagosomal membranes. LC3/GABARAP recruit cargo adaptors to link cargo to the autophagosome and engage tethering factors that bridge autophagosomes to lysosomes, thereby promoting autophagosome–lysosome fusion.

Macroautophagy (hereafter simply “autophagy”) is a conserved intracellular degradation pathway in which cytoplasmic components are sequestered into double-membrane autophagosomes and delivered to lysosomes for breakdown (Yang and Klionsky, 2010) (Figure 1A). In the central nervous system (CNS), autophagy serves as a critical quality-control mechanism that is essential for neuronal and glial survival. For example, mice with neuron-specific deletion of the essential autophagy gene *Atg5* (autophagy-related 5) develop progressive neurodegeneration accompanied by accumulations of cytoplasmic protein inclusions (Hara et al., 2006). Critically, this pathology occurs even in the absence of disease-associated mutant proteins, demonstrating that constitutive basal autophagy is required to prevent toxic buildup of spontaneously damaged proteins and organelles (Hara et al., 2006). Glial cells such as astrocytes likewise depend on autophagy, but they deploy it in complementary ways: astrocytes actively clear extracellular debris and protein aggregates (e.g., amyloid-β plaques), thereby protecting neighboring neurons (Kim et al., 2024; Potokar and Jorgacevski, 2025). Together, these observations establish that autophagy is not merely a housekeeping process but a fundamental mechanism for maintaining both cellular and network integrity in the brain.

The molecular machinery underlying autophagy is built around the autophagy-related 8 (ATG8) protein family, which in mammals consists of six paralogs: three microtubule-associated protein 1 light chain 3 (LC3) proteins (LC3A, LC3B and LC3C) and three gamma-aminobutyric acid type A receptor-associated protein (GABARAP) proteins (GABARAP, GABARAPL1 and GABARAPL2). All ATG8 proteins are conjugated (lipidated) onto nascent autophagosome membranes through the ubiquitin-like ATG12–ATG5–ATG16L1 conjugation system (Yang and Klionsky, 2010). Once anchored in the membrane (often referred to as LC3-II or GABARAP-II forms), these proteins serve as scaffolds to recruit other factors *via* short LC3-interacting region (LIR) motifs (Figure 1B). Despite sharing significant sequence homology (Lee and Lee, 2016), LC3 and GABARAP subfamilies have evolved specialized, non-redundant functions—a phenomenon we term the “ATG8 paralog code”. For instance, GABARAP and its close paralog GABARAPL1 (GABARAP-like 1) directly bind to a specific region of the γ2 subunit of GABA_A receptors, an interaction that promotes trafficking and surface stabilization of GABA_A receptor complexes at inhibitory synapses. In contrast, LC3 proteins lack the corresponding binding interface and do not participate in GABA_A receptor trafficking *via* direct interaction (Ye et al., 2021). This example illustrates a broader principle: LC3 and GABARAP subfamilies perform a molecular “division of labor” in autophagy, with distinct binding partners, substrates, and roles at different stages of the pathway.

This molecular specialization takes on added significance in the nervous system due to two unique features: extreme cellular polarization in neurons and functional complementarity between neurons and astrocytes. Autophagosome biogenesis in neurons is largely initiated in distal axons, often at presynaptic terminals or growth cone regions, rather than in the cell body. After formation, autophagosomes undergo robust retrograde transport along axonal microtubules back towards the soma, a journey driven by dynein motor proteins (Cheng et al., 2015). This spatial organization means that neurons must coordinate autophagy over extraordinarily long distances—a logistical challenge that does not exist in most cell types (Cai and Ganesan, 2022). By contrast, astrocytes execute autophagy in a more conventional intracellular layout but leverage it for specialized supportive roles: They maintain neurotransmitter and metabolic homeostasis, internalize extracellular protein aggregates, and even engulf entire synaptic structures for degradation (Parisi et al., 2024; Lee et al., 2025; Potokar and Jorgacevski, 2025). Thus, neurons and astrocytes share core autophagy machinery but deploy it in distinct, complementary ways that reflect their different anatomies and functions.

A third layer of specialization emerges at the subcellular level, particularly at synapses (Liang, 2019). Presynaptically, basal autophagy continuously removes aging synaptic vesicles and damaged organelles, maintaining neurotransmitter release. For example, the small GTPase Rab26 selectively marks vesicles for autophagic degradation (Binotti et al., 2015). Postsynaptically, autophagy regulates synaptic plasticity. During NMDA (N-methyl-D-aspartate) receptor-dependent long-term depression (LTD), autophagy is upregulated to facilitate internalization and degradation of AMPA (α-amino-3-hydroxy-5-methyl-4-isoxazolepropionic acid)receptors (Shehata et al., 2012; Shen et al., 2020). At inhibitory synapses, GABA_A receptor-associated protein (GABARAP) GABA_A receptor trafficking. When autophagy is disrupted, GABARAP becomes trapped in sequestosome 1 (p62/SQSTM1) aggregates, reducing receptor surface levels and causing network hyperexcitability (Hui et al., 2019; Ye et al., 2021). In summary, autophagy operates in a compartment-specific manner at synapses, with LC3 and GABARAP subfamilies playing distinct roles in maintaining synaptic proteostasis and circuit homeostasis.

Despite growing recognition of specialized autophagy pathways, three critical gaps remain. First, how LC3-GABARAP molecular specialization translates into functional differences between neurons and glia has not been systematically integrated. Second, cell-type-specific autophagy roles and their interplay in health and disease remain incompletely understood. Third, how compartment-specific defects drive distinct disease phenotypes—from aggregate accumulation in neurodegeneration to circuit dysfunction in epilepsy and autism—lacks a unified framework.

This review addresses these gaps by organizing current knowledge around three levels of specialization: (1) molecular LC3–GABARAP division of labor, (2) cell-type differences (neuronal *versus* astrocytic), and (3) compartmental roles (presynaptic *versus* postsynaptic). We discuss how dysregulation at each level drives specific central nervous system (CNS) disorders: impaired fusion and clearance cause neurodegeneration (Alzheimer’s and Parkinson’s), whereas synapse-specific defects disrupt circuit balance (autism and epilepsy). Finally, we highlight emerging tools—including spatial transcriptomics, live imaging, optogenetics, and patient-derived induced pluripotent stem cell (iPSC) models—that are illuminating the ATG8 code and enabling precision therapeutics.

By integrating molecular, cellular, and spatial dimensions, this review provides a framework for understanding how LC3 and GABARAP preserve neural proteostasis and how context-specific dysfunction causes disease. Our synthesis guides mechanistic research and targeted therapeutic development.

## 2 Molecular functions and divergence of LC3 vs. GABARAP in autophagy

All six mammalian ATG8 proteins - three LC3 isoforms and three GABARAP isoforms- are lipidated onto phagophore membranes. Recent genetic studies reveal they are not required for autophagosome nucleation or cargo capture but instead function primarily in later maturation step (Nguyen et al., 2016). For example, knockout of the entire ATG8 family in HeLa cells does not prevent autophagosome formation *per se*, yet fusion of autophagosomes with lysosomes is blocked. In practice, LC3 and GABARAP subfamilies perform a “division of labor”: LC3 proteins primarily promote early phagophore expansion and cargo loading, whereas GABARAPs drive autophagosome maturation, trafficking, and lysosomal fusion (Figure 1C).

**FIGURE 2:**
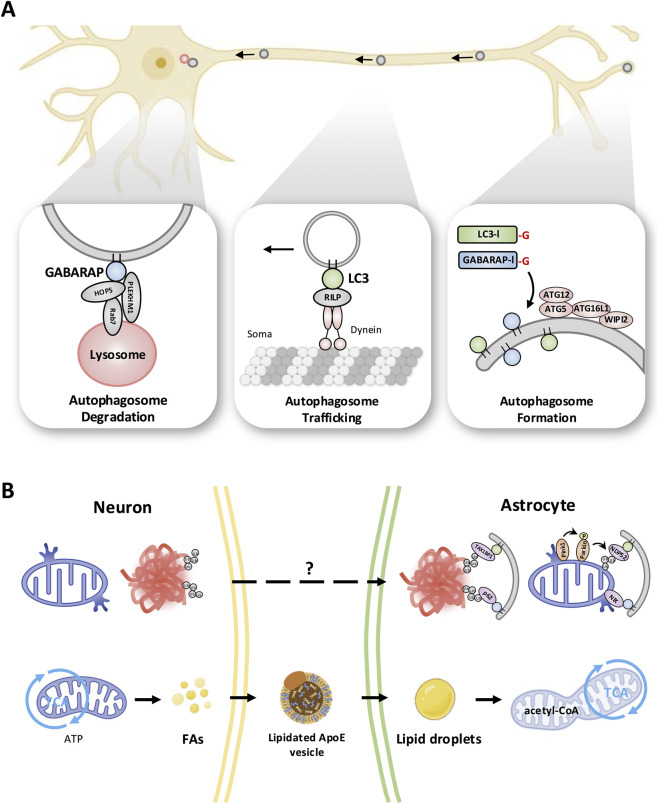
Molecular mechanisms of autophagy: LC3/GABARAP-mediated clearance and neuron-astrocyte crosstalk. **(A)** Neuron: In neurons, autophagosomes (grey) predominantly form in distal axons/presynaptic terminals and then undergo long-range retrograde transport to the soma for lysosomal degradation. LC3-decorated autophagosomes couple to dynein motors *via* the LC3-binding adaptor RILP for retrograde transport along microtubules, whereas GABARAP on mature autophagosomes promotes lysosomal fusion through PLEKHM1, Rab7, and the HOPS complex. Neuronal autophagy turns over damaged mitochondria by Parkin/PINK1-dependent mitophagy and ubiquitin-tagged protein aggregates by aggrephagy, with receptors such as NDP52 and NIX (mitophagy)and p62 and TAX1BP1 (aggrephagy)linking cargo to LC3 and GABARAP, and its efficiency declines with aging, leading to accumulation of undegraded cargo. **(B)** Astrocyte: Astrocytes exhibit robust autophagy that can be dynamically upregulated to support neurons. They internalize and degrade extracellular debris such as amyloid-β plaques and aggregates (red)by delivering them to lysosomes/autolysosomes, protecting neurons from proteotoxic stress. Astrocytic autophagy also mobilizes energy stores (e.g., lipid droplets) to nourish neurons under stress. Interplay with neurons: At the neuron–astrocyte interface (tripartite synapse, inset), astrocytes complement neuronal autophagy by clearing released protein aggregates and even whole damaged mitochondria from neurons (“transmitophagy”), highlighting a transcellular cooperation between neurons and astrocytes in maintaining brain homeostasis.

Indeed, Nguyen et al. (2016) showed that GABARAP subfamily members recruit the lysosome-tethering factor pleckstrin homology domain-containing family M member 1 (PLEKHM1) to facilitate autophagosome–lysosome fusion, whereas LC3 subfamily members play a much lesser role (Figure 2A). Genetic loss-of-function studies support ATG8 specialization, noting that GABARAP triple knockout markedly impairs autophagosome maturation and cargo clearance during starvation and Parkin-induced mitophagy, whereas LC3 triple knockout cells retain largely preserved (or only mildly delayed) autophagic flux, consistent with Nguyen et al. (2016). Mechanistically, PLEKHM1 fails to localize to autophagosomes in GABARAP-deficient cells, indicating that the final fusion step is stalled. Conversely, LC3 TKO cells still recruit cargo receptors (e.g., p62) and sequester mitochondria, albeit with somewhat slower phagophore growth. Quantitatively, GABARAP TKO cells accumulate autophagosomes (increased LC3-II levels) but show markedly reduced autophagosome-lysosome colocalization and impaired degradation of long-lived proteins. In contrast, LC3 TKO cells show near-normal rates of protein degradation and mitochondrial clearance, with only modest delays in autophagosome formation kinetics. These data confirm that GABARAPs are the primary drivers of autophagosome–lysosome fusion, whereas LC3 proteins mainly contribute to earlier elongation and cargo loading.

Mechanistically, these functional distinctions reflect different binding partners. Canonical cargo receptors (sequestosome 1 [p62/SQSTM1], NBR1, NDP52, etc.) use short LC3-interacting region (LIR) motifs to bind ATG8 proteins (Birgisdottir et al., 2013). PLEKHM1 is a notable example whose LIR motif is highly GABARAP-biased: structural analysis reveals PLEKHM1’s LIR binds GABARAP approximately 11-fold more tightly than LC3 (Rogov et al., 2017), effectively linking GABARAPs to the homotypic fusion and protein sorting (HOPS) complex-mediated fusion machinery. In fact, converting a standard LC3-interaction motif into a GABARAP-preferred “GABARAP-interaction motif” (GIM)—for example by introducing two valine residues—can switch adaptors like p62, FUN14 domain-containing 1 (FUNDC1), or focal adhesion kinase family interacting protein of 200 kDa (FIP200) from LC3-binding to GABARAP-binding. Thus, slight sequence changes in LIRs tune which ATG8 subfamily is engaged. In parallel, biochemical assays indicate intrinsic differences in membrane interaction. Zhang et al. (2023) demonstrated that LC3B’s N-terminal helix is essential for its lipidation and membrane association, whereas GABARAP’s N-terminus is not. Furthermore, Varela et al. (2025) report that GABARAP and GABARAPL1 uniquely drive vesicle tethering and membrane fusion *in vitro*, especially in the presence of ceramide, suggesting a mechanism for rapid phagophore expansion and sealing.

In neurons and glia, this molecular divergence has important implications. Neurons rely on high basal autophagy to clear damaged organelles and protein aggregates, and they express distinct pools of LC3 and GABARAP (Komatsu et al., 2006; Koike et al., 2013). Many mitophagy receptors (e.g., FUNDC1, BCL2 interacting protein 3 [BNIP3]/NIX) contain LIRs that can bind both LC3 and GABAR some neuronal adaptors and scaffolding proteins show clear preferences. For example, GABARAP associates with kinesin adaptors and soluble N-ethylmaleimide-sensitive factor attachment protein receptors (SNAREs) to support axonal autophagosome transport and fusion, while LC3 may predominantly engage synaptic cargo receptors (McEwan et al., 2015). Astrocytes and other glia may similarly exploit different LC3/GABARAP interactions to tune their autophagic flux under resting or stress conditions (Le Grand et al., 2011; Nikoletopoulou et al., 2017).

In summary, LC3 and GABARAP proteins have overlapping yet non-redundant autophagy roles: LC3-family ATG8 proteins mainly promote early membrane expansion and recruit ubiquitin-tagged cargo, whereas GABARAP-family ATG8 proteins orchestrate autophagosome maturation, trafficking, and lysosomal fusion. These differences are encoded by distinct LIR-binding preferences and regulatory motifs, and are increasingly recognized as critical for specialized autophagy functions in different cell types of the central nervous system (Figure 1).

## 3 Cell-type differences: Neuronal *versus* astrocytic autophagy

Neurons and astrocytes both rely on macroautophagy to maintain cellular health, but they do so in distinct ways reflecting their unique roles. Neurons are highly specialized, long-lived, post-mitotic cells that depend heavily AP, linking mitochondrial turnover to both subfamilies (Liu et al., 2012; Rogov et al., 2014). However, on autophagy to remove toxic or defective proteins and organelles in order to sustain neurotransmission and synaptic function (Karpova et al., 2025). In contrast, astrocytes use autophagy not only for their own homeostasis but also to support surrounding neurons by clearing extracellular protein aggregates and neuronal debris (Chung et al., 2013; Menzies et al., 2017). For example, astrocytic autophagy contributes to metabolic and redox homeostasis (e.g. mobilizing lipid droplets to fuel neurons under stress) (Ortiz-Rodriguez and Arevalo, 2020) and to clearance of neurotoxic aggregates (e.g. astrocytes internalize and degrade extracellular Aβ *via* autophagy) (Kim et al., 2024). Thus, while both cell types share core autophagy machinery, astrocytes engage autophagy through unique mechanisms that complement neuronal pathways.

Neuronal autophagy exhibits distinct spatial and temporal features. Autophagosome formation is enriched in distal axons and synapses, where damaged mitochondria and protein aggregates accumulate far from the soma. Although critical for synaptic proteostasis, neuronal autophagy is comparatively slow and declines with aging (Liang and Sigrist, 2018; Stavoe and Holzbaur, 2019). A critical finding is that neurons show a significant reduction in axonal autophagosome biogenesis with aging, leading to stalled isolation membranes and aberrant autophagosomes. This defect is linked to impaired regulation of WD repeat domain phosphoinositide-interacting protein 2 (WIPI2), a phosphoinositide-binding protein essential for autophagosome formation. Overexpressing WIPI2B rescues autophagosome formation in aged neurons. Thus, a WIPI2-dependent step in neuronal autophagy is especially vulnerable to aging (Stavoe et al., 2019; Stavoe and Holzbaur, 2020).

By contrast, astrocytes maintain more robust basal autophagy and can upregulate it dynamically (Kim et al., 2024). For example, astrocytes dynamically upregulate autophagy in response to amyloid-β (Aβ). Aβ exposure induces LC3B and sequestosome 1 (p62/SQSTM1) expression in astrocytes but not neurons, accelerating Aβ clearance. Functionally, when astrocytic autophagy is inhibited (e.g., by LC3B knockdown in Alzheimer’s disease model mice), Aβ clearance is impaired, leading to larger amyloid plaques and worsened cognitive deficits. Likewise, stimulating astrocytic autophagy by mechanistic target of rapamycin complex 1 (mTORC1) inhibition reduces Aβ secretion and promotes its degradation. These findings demonstrate that astrocytes, unlike neurons, can dynamically upregulate autophagy to clear extracellular protein aggregates such as Aβ.

Aside from these homeostatic roles, neurons and astrocytes interact through autophagic pathways in aging and disease. A striking example is transmitophagy (Lampinen et al., 2022): under physiological conditions, astrocytes engulf and degrade neuronal mitochondria. This intercellular mitophagy pathway is enhanced in disease (e.g., Alzheimer’s disease models) and may compensate when neuronal mitophagy is compromised. Conversely, recent work shows that neurons can transfer entire autophagosomes to astrocytes for disposal—a novel clearance route that bypasses slow axonal transport. Furthermore, astrocytes clear other neuronal debris: they internalize extracellular α-synuclein or tau and degrade them *via* autophagy, though this capacity declines in senescent or apolipoprotein E4 (APOE4)-expressing astrocytes (Lee et al., 2010; Simonovitch et al., 2016). Notably, astrocytic mitophagy is more rapid and less spatially restricted than neuronal mitophagy (Maday et al., 2012; Davis et al., 2014), reflecting faster PTEN-induced kinase 1 (PINK1)-dependent pathways in astrocytes. In sum, neuron-specific cargo (synaptic vesicles, organelles)and astrocyte-specific roles (aggresome clearance, transmitophagy) lead to distinct cargo selectivity in each cell type (Howden et al., 2025; Karpova et al., 2025) (Figure 2B).

Together, these differences demonstrate complementary autophagy functions in neurons and astrocytes. Neuronal autophagy supports long-range intracellular proteostasis but declines with aging due to WIPI2 dysfunction (Stavoe et al., 2019). In contrast, astrocytic autophagy provides metabolic support and clears extracellular and transcellular waste, including Aβ and neuronal mitochondria (Lampinen et al., 2022; Kim et al., 2024). These complementary functions create interdependence: impairing autophagy in one cell type compromises the other, emphasizing the critical importance of cell-type-specific therapeutic strategies. For example, neuronal autophagy defects drive neurodegeneration, whereas astrocytic autophagy deficits (including APOE4-mediated impairment) exacerbate Aβ pathology (Stavoe et al., 2019; Kim et al., 2024).

It is important to note that much of our mechanistic understanding derives from rodent models and immortalized cell lines. While these systems have been invaluable for dissecting molecular pathways, key differences exist: human neurons are larger with more complex dendritic arbors, human astrocytes show greater morphological and functional diversity, and aging timescales differ dramatically. Induced pluripotent stem cell (iPSC)-derived models bridge this gap but have limitations, including immature phenotypes and lack of circuit context. Therefore, findings from animal models must be cautiously extrapolated to human disease, and validation in human tissue or advanced organoid systems is essential (Figure 2).

## 4 Compartmental functions: Pre- *versus* postsynaptic autophagy

Autophagy in neurons is highly compartmentalized, reflecting the distinct proteostatic needs of pre- and postsynaptic sites. This spatial specialization is now well established from live imaging and ultrastructural studies in mammalian neurons (Stavoe and Holzbaur, 2019).

### 4.1 Presynaptic autophagy

Presynaptic autophagy regulates the turnover of synaptic vesicle (SV) pools, active-zone proteins, and presynaptic mitochondria, thereby maintaining release competence and metabolic homeostasis (Okerlund et al., 2017; Stavoe and Holzbaur, 2019; Wosnitzka et al., 2025). In resting or low-activity synapses, a basal level of autophagy continually recycles aged or damaged SV proteins, and during periods of intense activity or oxidative/metabolic stress, this process is rapidly upregulated (Hernandez et al., 2012; Soukup and Verstreken, 2017; Hoffmann et al., 2019).

Pioneering work in cultured mouse hippocampal neurons demonstrated that disabling the giant active-zone scaffold Bassoon triggers local autophagy: genetic deletion of Bassoon induces robust autophagosome formation in axon terminals with concomitant SV depletion (Okerlund et al., 2017). Mechanistically, Bassoon directly binds the autophagy E3-like protein ATG5 and inhibits premature autophagy; thus, Bassoon loss releases this brake, allowing SV pools to be funneled into LC3-positive phagophores. This establishes that the LC3 conjugation machinery operates at presynapses for selective vesicle turnover, and that synaptic integrity depends on tight local regulation of autophagy (Okerlund et al., 2017; Yang et al., 2022).

More recent studies in *C. elegans* and mammalian cultured neurons revealed that ATG9 vesicle trafficking couples the SV cycle to autophagosome biogenesis, providing a mechanistic link between activity-dependent endocytosis and presynaptic autophagy (Yang et al., 2022). Disease-associated mutations in endocytic proteins (e.g., Synaptojanin1, implicated in familial Parkinson’s disease) disrupt this ATG9-SV coupling, impairing activity-induced autophagy at synapses (Yang et al., 2022). Additionally, *in vitro* and *ex vivo* studies showed that Endophilin-A integrates Ca^2+^ influx with activity-induced presynaptic autophagy, and this function is perturbed by a Parkinson’s-risk mutation (R183Q) (Bademosi et al., 2023). Complementing these findings, the small GTPase Rab26 marks specific SV subsets for autophagic degradation: Rab26-positive SV clusters attract ATG16L1/LC3 and are routed into preautophagosomal structures, suggesting a selective pathway for excess SV disposal (Binotti et al., 2015).

Presynaptic autophagosomes typically originate in distal axons and undergo retrograde transport toward the soma for lysosomal clearance, a transport logic shared with mitophagosomes (Maday et al., 2012; Maday and Holzbaur, 2014; Stavoe and Holzbaur, 2019). Damaged presynaptic mitochondria are cleared *via* PINK1/Parkin-mediated mitophagy: damaged organelles recruit PINK1/Parkin, become engulfed by LC3/GABARAP membranes, and dynein–Snapin motors mediate retrograde transport of mitophagosomes to prevent ROS buildup and synaptic degeneration (Narendra et al., 2008; Cai et al., 2012; Stavoe and Holzbaur, 2019).

Both insufficient and excessive presynaptic autophagy are deleterious: too little autophagy causes accumulation of damaged SVs and organelles, whereas hyperactivation (e.g., in Bassoon-deficient synapses) depletes functional SV pools and impairs neurotransmitter release (Hernandez et al., 2012; Okerlund et al., 2017; Wosnitzka et al., 2025). This dual requirement underscores the importance of precise local regulation.​

### 4.2 Postsynaptic autophagy

Postsynaptic autophagy modulates receptor and scaffold turnover in an activity-dependent manner, contributing to synaptic plasticity (Shehata et al., 2012; Kallergi et al., 2022; Janusz-Kaminska et al., 2024). In cultured rat hippocampal neurons, NMDA receptor activation (a cellular model for LTD) induces postsynaptic autophagy, evidenced by a rapid increase in dendritic LC3-II and a rise in LC3-positive puncta within dendritic spines; this NMDA-driven autophagy promotes the degradation of AMPA receptor subunits (e.g., GluA1), linking autophagy activation to activity-dependent receptor turnover (Shehata et al., 2012). Consistent with an autophagy-promoting role, inhibiting autophagy genetically (ATG7 knockdown) or pharmacologically (autophagic flux inhibition) preserves surface AMPARs and prevents LTD induction, indicating that activation of postsynaptic autophagy is required for this form of synaptic weakening (Shehata et al., 2012). Importantly, recent *in vivo* studies in mice confirmed that dendritic autophagosomes selectively sequester and degrade postsynaptic density components during LTD, and LTD is impaired in autophagy-deficient (Atg5 conditional knockout)mice (Kallergi et al., 2022). Emerging work suggests that a sequential pathway involving Rab11, ATG9A, and LC3 initiates postsynaptic autophagosome formation at dendritic spines during synaptic plasticity (Puri et al., 2013; Kallergi et al., 2022; Janusz-Kaminska et al., 2024). Together, these data establish a necessary role for postsynaptic autophagy in activity-dependent weakening of excitatory synapses (Kallergi et al., 2022; Janusz-Kaminska et al., 2024).

GABARAP family proteins are particularly implicated at inhibitory postsynapses. As described in the Introduction, GABARAP and GABARAPL1 directly bind the γ2 subunit of GABA_A receptors and promote receptor trafficking and surface stability (Chen et al., 2000; Kanematsu et al., 2007). Critically, in autophagy-deficient models (Atg7 conditional knockout in GABAergic interneurons), sequestration of GABARAP in p62/SQSTM1 aggregates prevents normal GABA_A receptor trafficking, leading to reduced inhibitory synaptic strength and network hyperexcitability (Hui and Tanaka, 2019). By contrast, robust evidence for a direct GABARAP role in AMPA/NMDA receptor endocytosis is limited; any influence on ionotropic glutamate receptors appears indirect *via* postsynaptic scaffolds or general proteostasis (Stavoe and Holzbaur, 2019; Wosnitzka et al., 2025).

### 4.3 Mechanistic distinctions between LC3 and GABARAP at synapses

**FIGURE 3:**
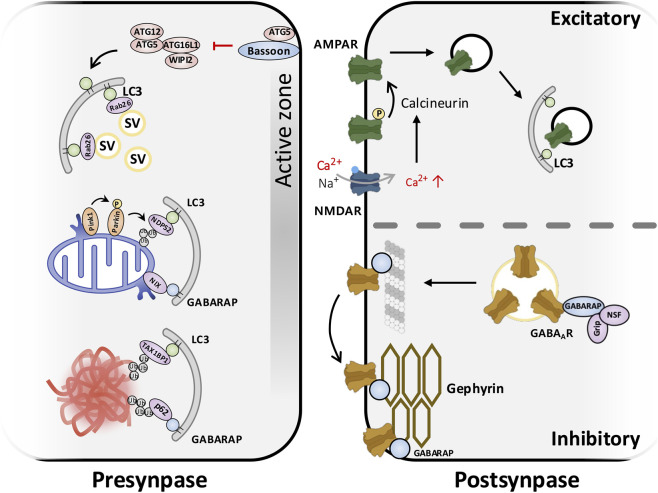
Compartment-specific autophagy at synapses. Presynaptic (left panel): At axon terminals, basal autophagy (LC3-positive membranes) continuously eliminates a subset of aging synaptic vesicles and worn-out presynaptic proteins. For instance, the GTPase Rab26 selectively targets synaptic vesicles to LC3-positive autophagic membranes. Large scaffolds like Bassoon normally suppress excessive autophagy at the active zone by sequestering ATG5 and limiting recruitment of the ATG12–ATG5–ATG16L1–WIPI2 LC3-conjugation machinery; when Bassoon is lost, this complex accumulates at the presynapse, autophagosomes rapidly form, and synaptic vesicle pools become depleted. Excitatory synapses (upper panel): In dendritic spines, autophagy is engaged during synaptic plasticity. Upon NMDA receptor activation during long-term depression (LTD), LC3-labeled autophagosomes sequester internalized AMPA receptors and associated scaffold proteins, facilitating their removal from the postsynaptic membrane. This autophagy-dependent receptor clearance contributes to the persistent weakening of excitatory synapses, and mice or neurons lacking core autophagy genes (e.g., ATG7 knockout) fail to degrade these postsynaptic cargos, resulting in impaired LTD and excess surface AMPA receptors. Inhibitory synapses (lower panel): GABARAP family proteins at inhibitory synapses bind GABA_A receptor subunits and the scaffold gephyrin, and interact with trafficking factors such as GRIP and NSF, promoting proper delivery and stabilization of GABA_A receptors at the postsynaptic membrane. If autophagy is compromised, GABARAP and GABARAPL1/2 become trapped in p62-positive aggregates, preventing them from escorting GABA_A receptors to gephyrin clusters. Consequently, inhibitory receptors fail to remain concentrated at synapses, reducing inhibitory tone and potentially leading to network hyperexcitability (relevant to epilepsy and autism models). In sum, LC3- and GABARAP-dependent pathways operate locally in pre- and postsynaptic compartments to turn over specific substrates and keep neurotransmission in balance, and their dysregulation at synapses can drive neurological disease.

Accumulating evidence indicates compartmental and functional biases between LC3 and GABARAP subfamilies at synapses (Stavoe and Holzbaur, 2019; Wosnitzka et al., 2025). LC3 puncta are readily observed in dendritic spines after long-term depression (LTD)-like stimulation, consistent with roles in cargo sequestration and degradation of excitatory receptor complexes (Shehata et al., 2012; Kallergi et al., 2022). In contrast, GABARAP shows conspicuous enrichment at inhibitory synapses and the axon initial segment, consistent with specialized roles in inhibitory receptor trafficking and in late steps of autophagosome maturation and fusion (Koike et al., 2013; Hui et al., 2019). Moreover, as discussed in Section 1, GABARAP family members preferentially recruit tethering factors such as PLEKHM1 to support autophagosome–lysosome fusion, complementing LC3’s broader roles in phagophore expansion and cargo capture (Tabata et al., 2010; McEwan et al., 2015) (Figure 3).

### 4.4 Model limitations and human relevance

Most mechanistic studies of synaptic autophagy derive from cultured rodent neurons, invertebrate models (*C. elegans, Drosophila*), or acute brain slices from young rodents. While these systems have been invaluable for dissecting molecular pathways, key differences exist in human synapses: larger neurons with more complex dendritic arbors, distinct synaptic protein composition (particularly at inhibitory synapses), and dramatically different activity patterns and aging timescales. The extent to which specific findings—such as the Bassoon-ATG5 interaction, Rab26-mediated synaptic vesicle sorting, or Rab11-ATG9A sequences—generalize to human synapses *in vivo* remains to be fully established.

Advanced human-derived systems are beginning to address this gap: patient iPSC-derived neurons carrying autophagy-gene mutations (e.g., WDR45/WIPI4 in β-propeller protein-associated neurodegeneration [BPAN]) show impaired autophagic flux and shortened axons, supporting the translational relevance of rodent mechanisms (Huang et al., 2025). Going forward, integrated studies across human iPSC-derived neuronal cultures, cerebral organoids with functional synapses, and *postmortem* brain tissue will be essential to determine which synaptic autophagy mechanisms are conserved and which are species-specific. In parallel, direct reprogramming of adult somatic cells (e.g., fibroblasts) into induced neurons or induced astrocytes (iNs/iAs) can complement iPSC models by retaining donor age-associated signatures and enabling matched neuron–astrocyte co-cultures to probe human-specific autophagy crosstalk (Mertens et al., 2015; Tang et al., 2017; Quist et al., 2022).

## 5 Disease relevance: Compartmental and cell-type specific pathogenesis

### 5.1 Alzheimer’s disease (AD)

Autophagic flux is disrupted very early in AD, leading to accumulation of autophagic vacuoles (AVs)in dystrophic neurites (Boland et al., 2008). Indeed, LC3-positive autophagosomes filled with Aβ and amyloid precursor protein (APP) metabolites build up within swollen axons prior to extracellular plaque formation (Tammineni et al., 2017). This suggests that neurons fail to efficiently fuse autophagosomes with lysosomes–possibly due to impaired GABARAP-mediated trafficking of fusion machinery or other endolysosomal defects–causing toxic protein aggregates to accumulate.

Astrocytic autophagy also plays a critical role in amyloid clearance. A recent study showed that astrocyte-specific overexpression of LC3B significantly accelerates Aβ clearance and rescues cognitive function in APP/presenilin 1 (PS1) transgenic mice (Kim et al., 2024). Conversely, blocking autophagy in astrocytes (e.g., *via* LC3B or p62 knockdown) exacerbates amyloid plaque deposition and neuroinflammation (Kim et al., 2024). Thus, in AD, the interplay is multifaceted: neuron-intrinsic LC3/GABARAP deficits allow intracellular Aβ/APP fragments to accumulate, while astrocytic autophagy capacity determines how effectively extracellular Aβ aggregates are removed (Kim et al., 2024).

Notably, emerging therapeutics target these pathways. Sigma-1 receptor (Sig1R) agonists that boost LC3 lipidation and autophagic flux are under clinical evaluation to mitigate early AD progression (Prasanth et al., 2021). Such Sig1R activators (e.g., ANAVEX2-73, AF710B) induce autophagy and have shown neuroprotective effects in preclinical models, underscoring the potential of LC3 upregulation in early AD (Hall et al., 2018; Prasanth et al., 2021).

### 5.2 Parkinson’s disease (PD)

Parkinson’s disease (PD)is characterized by α-synuclein (α-syn) aggregation and dopaminergic neuron loss, with multiple autophagy-lysosome pathway genes implicated. For example, mutations in GBA1 (encoding lysosomal glucocerebrosidase) and VPS35 (encoding vacuolar protein sorting 35, a retromer component) are common genetic risk factors for PD that compromise lysosomal clearance and autophagy, accelerating α-syn accumulation (Schondorf et al., 2014; Zavodszky et al., 2014). In neurons, LC3-dependent mitophagy *via* the PINK1/Parkin pathway is critical for mitochondrial quality control in long axons. Loss of Parkin or PINK1 reduces LC3 recruitment to damaged mitochondria, leading to defective mitophagy and axonal degeneration (Basak and Holzbaur, 2025).

A recent study identified a novel stimulator of interferon genes (STING)–conjugation of ATG8 to single membranes (CASM)–GABARAP pathway at lysosomes by which lysosomal stress aberrantly activates leucine-rich repeat kinase 2 (LRRK2), impairing autophagosome–lysosome fusion (Bentley-DeSousa et al., 2025). Consistently, PD-linked LRRK2 mutations alter GABARAP phosphorylation and disrupt autophagosome tethering/fusion *via* this pathway (Chu, 2011; Esteves and Cardoso, 2017; Boecker and Holzbaur, 2021; Pang et al., 2022).

Additionally, PD pathology involves astroglia: astrocyte-targeted autophagy enhancers (e.g. lithium, which induces autophagy) protect dopaminergic neurons against MPP^+^ toxicity by boosting astrocytic LC3-dependent autophagic flux (Shen et al., 2016). In contrast, impaired astrocytic autophagy exacerbates neuroinflammation and oxidative stress in the midbrain (Ortiz-Rodriguez and Arevalo, 2020). In sum, PD appears to involve both neuronal and astrocytic LC3/GABARAP dysfunction: neuron-intrinsic autophagy defects allow α-syn and damaged mitochondria to accumulate, while deficient astrocytic autophagy lowers extracellular buffering capacity and intensifies neuronal stress.

### 5.3 Neurodevelopmental disorders (autism spectrum disorder, ASD)

Growing evidence links autophagy deficits to autism spectrum disorder (ASD). Mutations in tuberous sclerosis complex 1 (*TSC1*)/tuberous sclerosis complex 2 (*TSC2*) (negative regulators of mTOR) and phosphatase and tensin homolog (*PTEN*) (phosphatidylinositol 3-kinase [PI3K] pathway inhibitor)—which hyperactivate mTOR and thereby downregulate autophagy—are associated with syndromic ASD (Tang et al., 2014). Critically, deleting *Atg7* selectively in forebrain GABAergic interneurons of adolescent mice causes social behavior deficits and hyperactivity reminiscent of ASD (Hui et al., 2019). As discussed in Section 3, autophagy deficiency in these neurons causes GABARAP sequestration in p62-positive aggregates, preventing normal GABA_A receptor trafficking and leading to reduced inhibitory signaling and cortical hyperexcitability. Thus, a cell-autonomous loss of LC3/GABARAP flux at GABAergic synapses can lead to circuit-level excitation/inhibition imbalance underlying ASD-like behaviors.

Similarly, loss of *TSC1/2* or direct autophagy-related gene (*Atg*) knockout in excitatory neurons disrupts synaptic protein turnover, leading to excess excitatory synapse density and epileptiform activity (Tang et al., 2014). These models underscore that synapse-specific autophagy—particularly GABARAP-mediated trafficking of inhibitory receptors—is crucial for normal social behavior and neural network balance during development.

### 5.4 Epilepsy

Seizures robustly engage neuronal autophagy pathways in an activity-dependent manner. Acute seizure episodes increase the LC3-II/LC3-I ratio and decrease p62 levels in affected brain regions, indicating upregulated autophagic flux following intense neuronal firing (Li et al., 2018). This autophagy surge is thought to be a protective response to clear damaged proteins or mitigate excitotoxic stress.

GABARAPs also play a role in epilepsy pathophysiology. As noted above, GABARAP is required for normal GABA_A receptor trafficking to inhibitory synapses (Hui et al., 2019; Ye et al., 2021). In chronic epilepsy models, knockdown of GABARAP family members leads to instability of GABA_A receptor clusters at synapses, reducing inhibitory tone and increasing network excitability, thereby lowering seizure threshold (Hui et al., 2019).

Astrocyte autophagy status is likely to modulate seizure susceptibility: astrocytic glutamate transporters are known to control excitotoxic seizures, and defective autophagy/mitophagy can promote interleukin-1β (IL-1β) hypersecretion *via* accumulation of damaged mitochondria, a cytokine that has well-established pro-convulsant actions (Al-Kuraishy et al., 2025; Lee et al., 2025). Thus, impaired LC3/GABARAP function in either neurons or astrocytes can predispose circuits to hyperexcitability. For example, reduced neuronal LC3 lipidation may slow the removal of overstimulated AMPA receptors or damaged organelles after intense activity, prolonging excitatory signaling (Shehata et al., 2018). Conversely, defective GABARAP-mediated trafficking of GABA_A receptors (as seen in the ASD model above) diminishes inhibition and can precipitate seizure activity. Enhancing autophagy—e.g., *via* mTOR inhibitors like rapamycin—has shown promise in some models by restoring the balance of excitatory/inhibitory receptor turnover and reducing spontaneous seizures (Li et al., 2018; Hui et al., 2019; Ye et al., 2021).

### 5.5 Other disorders (ALS, Huntington’s disease, etc.)

In amyotrophic lateral sclerosis (ALS), misfolded protein aggregates (superoxide dismutase 1 [SOD1], TAR DNA-binding protein 43 [TDP-43], etc.) accumulate in motor neurons in part due to autophagy failure and defective protein clearance (Barmada et al., 2014; Taylor et al., 2016). Remarkably, this can be aggravated non-cell-autonomously: ALS patient-derived astrocytes secrete factors that impair motor neuron autophagy and proteostasis (Madill et al., 2017). In co-culture experiments, motor neurons exposed to conditioned medium from ALS astrocytes exhibit decreased LC3-II levels and accumulate p62 and misfolded proteins, an effect alleviated by autophagy activators. GABARAP dysfunction may contribute to ALS pathophysiology by disrupting GABAergic inhibitory synapses in the spinal cord, potentially promoting motor circuit hyperreflexia (Kneussel et al., 2000; Tseng et al., 2015; Rudnick et al., 2017; Amin et al., 2020).

Huntington’s disease (HD) is another example where autophagy is pivotal: neuronal macroautophagy normally helps clear mutant huntingtin (mHTT) protein aggregates (Ravikumar et al., 2004; Martinez-Vicente et al., 2010). If neuronal autophagy is impaired, mHTT fragments accumulate, causing synaptic dysfunction and neurodegeneration. Notably, recent findings indicate that reactive astrocytes can bolster proteostasis and protect neurons in HD by enhancing aggregate clearance (Abjean et al., 2023). For instance, activation of the Janus kinase 2 (JAK2)–signal transducer and activator of transcription 3 (STAT3) pathway in astrocytes increases their lysosomal degradation capacity and reduces mHTT aggregate load in neighboring striatal neurons. Astrocytic autophagy (and related proteolytic mechanisms) aids in degrading extracellular or synaptic debris, including released mHTT fragments, thereby curbing the spread of toxic protein species.

LC3/GABARAP dysfunction contributes to CNS disorders in a compartment- and cell-type-specific manner (Table 1). Neuron-intrinsic defects in LC3-dependent autophagy promote toxic protein accumulation and synapse loss, as observed in Alzheimer’s and Parkinson’s disease models. By contrast, synapse-restricted defects—such as impaired GABARAP-mediated GABA_A receptor trafficking—disrupt excitation–inhibition balance and drive network hyperexcitability, mechanisms relevant to autism spectrum disorders and epilepsy. Beyond neurons, glial autophagy failure exacerbates pathology in a non–cell-autonomous fashion. Defective astrocytic autophagy impairs extracellular aggregate clearance and alters inflammatory tone in models of AD, ALS, and HD. Collectively, these findings position LC3- and GABARAP-linked autophagy pathways in neurons and glia as distinct but complementary therapeutic targets for preserving synaptic integrity and network stability across neurodegenerative and neurodevelopmental disorders.

**TABLE 1:** Key human-relevant studies on LC3/GABARAP function in CNS disorders.

Disease	Cell type	Model system	LC3/GABARAP alteration	Key references
Alzheimer’s disease	Neurons, Astrocytes	*Postmortem* brain tissue; APP/PS1 transgenic mice; patient iPSC-derived astrocytes	↑ LC3-positive autophagic vacuoles in neurons	Nixon et al. (2005)
			↑ LC3B in astrocytes improves Aβ clearance	Kim et al. (2024)
Parkinson’s disease	Dopaminergic neurons	*Postmortem* brain tissue; GBA1-mutant patient iPSC neurons; LRRK2 mouse models	↓ GABARAP/LC3 isoforms	Tanji et al. (2011)
			Impaired GABARAP-mediated fusion	Schondorf et al. (2014), Bentley-DeSousa et al. (2025)
Autism spectrum disorder	GABAergic interneurons	ATG7 conditional knockout mice; TSC1/2 patient iPSC neurons	GABARAPL2 sequestration in p62 aggregates	Hui et al. (2019)
			Reduced GABA_A receptor surface expression	Tang et al. (2014)
Epilepsy	Excitatory/Inhibitory neurons	Seizure-induced rodent models; human epileptic tissue	↑ LC3-II/LC3-I ratio (acute)	Li et al. (2018)
			↓ GABARAP at inhibitory synapses (chronic)	Hui et al. (2019)
Amyotrophic lateral sclerosis	Motor neurons, Astrocytes	Patient iPSC-derived motor neurons and astrocytes; SOD1 mouse models	↓ LC3-II in motor neurons exposed to ALS astrocyte factors	Madill et al. (2017)
			↑ p62 aggregates	Rudnick et al. (2017)
Huntington’s disease	Striatal neurons, Astrocytes	*Postmortem* brain tissue; mHTT transgenic mice; patient iPSC neurons	Impaired LC3-mediated mHTT clearance	Martinez-Vicente et al. (2010)
			Astrocytic JAK2-STAT3 enhances lysosomal degradation	Abjean et al. (2023)

This table summarizes disease condition, cell type studied, model system, observed LC3/GABARAP, alterations, and key references for major human-relevant findings discussed in this review. ↑ indicates increase, ↓ indicates decrease.

## 6 Experimental tools and future directions

Recent years have seen a surge in innovative methodologies to interrogate LC3/GABARAP-mediated autophagy in neural cells. These approaches span multi-omics profiling, live imaging and biosensors, activity-dependent analyses, targeted manipulations, and translational human models. Below, we organize key experimental tools and future directions under cohesive themes, emphasizing advances from the relevant findings. Importantly, many of these tools are accelerating the translation of basic autophagy insights into the context of human brain health and disease.

### 6.1 Omics approaches for LC3/GABARAP research

Spatial transcriptomics and single-cell genomics have enabled high-resolution mapping of autophagy-related gene expression in intact brain tissues. For example, spatial RNA profiling in multiple sclerosis lesion areas revealed region-specific autophagy deficits: core regions of chronic active lesions showed significantly reduced expression of key *Atg* genes (including LC3/GABARAP-family members), inversely correlated with intense local inflammation (Misrielal et al., 2022). Similarly, in Parkinson’s disease (PD) cortex, spatial transcriptomics of Lewy body-bearing neurons identified a conserved molecular signature of dysfunction that includes downregulation of lysosomal and autophagy genes in neurons harboring α-synuclein aggregates. Goralski et al. (2024) showed that excitatory cortical neurons with Lewy pathology have reduced expression of genes related to synaptic function, mitochondria, endolysosomes, and proteasomes, indicating that protein aggregates are linked to impaired autophagy-lysosomal function. Such transcriptomic atlases offer unprecedented insight into cell-type and region-specific autophagy states *in situ*, guiding hypotheses on where autophagy support might be most needed in disease.

**FIGURE 4:**
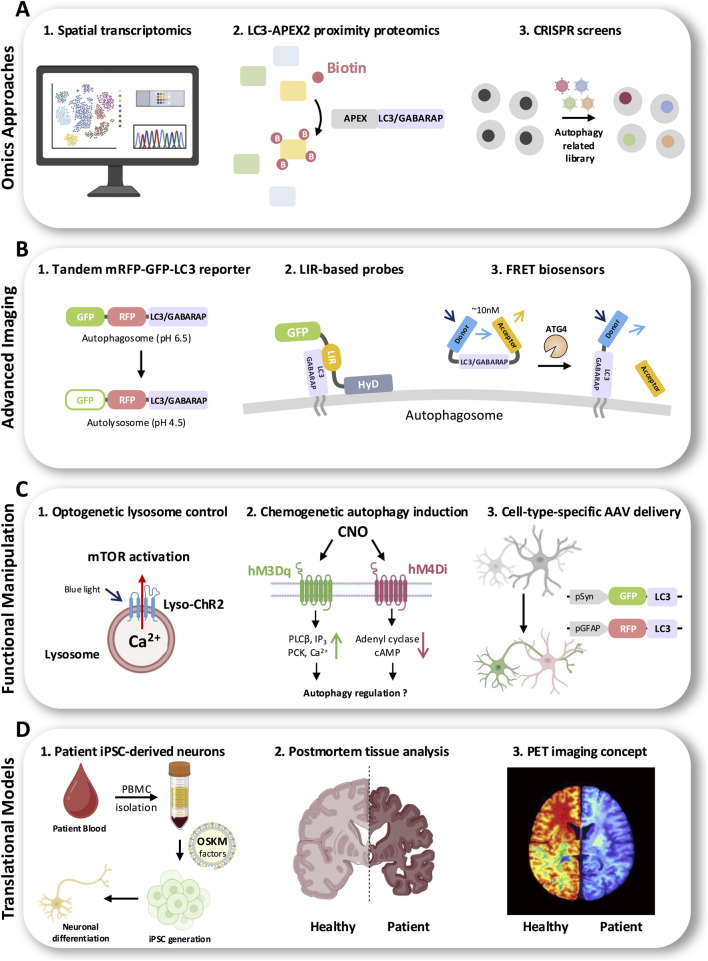
Emerging methodologies to interrogate LC3/GABARAP-dependent autophagy in the nervous system. Schematic overview of complementary experimental platforms spanning discovery, live readouts, causal manipulation, and translation. **(A)** Omics approaches: Spatial transcriptomics enables anatomical, cell-type-resolved mapping of autophagy and lysosome-related gene programs across tissues. LC3-APEX2 proximity proteomics uses APEX-catalyzed biotinylation to label and identify proteins in the immediate vicinity of LC3/GABARAP-positive membranes, capturing context-dependent interactomes. Pooled CRISPR screens using autophagy-focused libraries systematically identify genetic regulators of autophagy and ATG8-dependent processes. **(B)** Live imaging and biosensors: The tandem mRFP–GFP–LC3 reporter distinguishes autophagosomes from autolysosomes based on pH-dependent GFP quenching in acidic compartments, enabling autophagic flux measurements. LIR-based probes (fluorescent LC3-interacting region modules) bind LC3/GABARAP on autophagosomal membranes to visualize ATG8-decorated structures in cells. FRET biosensors report ATG8 processing or state changes (e.g., ATG4-dependent cleavage/processing) *via* donor–acceptor proximity, allowing real-time readouts of autophagy-associated enzymatic steps. **(C)** Functional manipulation: Optogenetic lysosome modulation (e.g., Lyso-ChR2) uses light to alter lysosomal ion fluxes (Ca^2+^) and downstream signaling such as mTOR activity. Chemogenetic approaches (DREADDs hM3Dq/hM4Di) activated by CNO engage distinct second-messenger pathways (PLCβ/IP_3_/Ca^2+^ or adenylyl cyclase/cAMP) to tune autophagy-related signaling. Cell-type-specific AAV delivery of LC3 reporters (e.g., neuronal pSyn vs. astrocytic pGFAP promoters) enables selective visualization or manipulation in defined neural populations. **(D)**Translational human systems: Patient-derived iPSC neurons generated from blood PBMCs *via* OSKM reprogramming provide genotype-matched cellular models for mechanistic studies and therapeutic testing. *Postmortem* brain analyses enable direct comparison of autophagy/lysosome markers in healthy *versus* patient tissue. Conceptual PET imaging strategies illustrate the goal of noninvasively quantifying autophagy/lysosomal pathway activity in living subjects. APEX2, enhanced ascorbate peroxidase 2; LIR, LC3-interacting region; FRET, Förster resonance energy transfer; AAV, adeno-associated virus; iPSC, induced pluripotent stem cell; PET, positron emission tomography; DREADD, designer receptor exclusively activated by designer drugs; CNO, clozapine-N-oxide; ChR2, channelrhodopsin-2. OSKM; OCT4 (POU5F1), SOX2, KLF4, and c-MYC (the “Yamanaka factors”); PBMC; Peripheral Blood Mononuclear Cells,; pSyn: synapsin promoter (often written as hSyn or Syn1 promoter, neuron-preferential); pGFAP: glial fibrillary acidic protein promoter (astrocyte-associated).

Complementing transcriptomics, proteomic approaches have expanded our view of the LC3/GABARAP interactome and autophagic cargo in neurons (Figure 4A). Cutting-edge proximity labeling proteomics uses engineered peroxidases (APEX2) or biotin ligases (TurboID) fused to LC3 or its binding partners to catalogue the molecular microenvironment of autophagosomes. In a landmark study, an LC3B-APEX2 proximity proteomics strategy identified hundreds of proteins in the vicinity of LC3 under basal conditions, including unexpected lysosomal and endosomal factors, ESCRT-III subunits, and specific cargo receptors (Le Guerroue et al., 2017). Another study developed an LC3B-APEX “autophagosome content” mouse model that enables *in vivo* biotinylation and identification of autophagosome cargo. Using this system, IL-7 receptor-α (IL7Rα) was identified as a selective autophagy cargo in T cells, showing that autophagic degradation of IL7Rα fine-tunes cytokine signaling during T-cell activation and providing a proof-of-concept for LC3-APEX2 in mammals (Zhou et al., 2022).

In neurons, integrated proteomics of human iPSC-derived neurons and mouse brains with autophagy gene knockouts has defined a broad “autophagy degradome” – the spectrum of proteins and organelle components that accumulate when autophagy is blocked. Strikingly, this approach revealed that neuronal autophagy constitutively turns over proteins from the ER, Golgi, endosomes, mitochondria and synaptic vesicles, among others. It also led to the discovery of new autophagy cargo receptors in neurons: for instance, A-kinase anchoring protein 11 (AKAP11) was identified as an autophagic receptor responsible for constantly clearing the PKA-RI regulatory subunit of protein kinase A, thereby coupling autophagy to cAMP-PKA signaling homeostasis (Deng et al., 2021; Zhou et al., 2024). This finding highlights a newly recognized role for autophagy in controlling neuronal excitability and transcription *via* degradation of specific signaling complexes.

Additionally, large-scale CRISPR-Cas9 genetic screens are being applied to autophagy (Liang and Corn, 2022) (Figure 4A). Genome-wide CRISPR knockout libraries have pinpointed both core autophagy factors and modulators (e.g. lipid remodeling enzymes, ubiquitin ligases) essential for LC3 processing and autophagosome biogenesis (Morita et al., 2018; Shoemaker et al., 2019). Such functional genomics approaches are elucidating the unique genetic dependencies of autophagy in neurons *versus* other cell types. Together, multi-omics tools are furnishing a systems-level understanding of LC3/GABARAP pathways in the brain and revealing novel therapeutic target.

### 6.2 Advanced imaging and biosensors

Imaging approaches have grown increasingly sophisticated, allowing real-time visualization of autophagy dynamics in living neural cells and even *in vivo* (Figure 4B). Transgenic mice expressing dual-fluorescent LC3 reporters have been pivotal for spatiotemporal tracking of autophagic flux in the brain (Castillo et al., 2017). For example, the tandem mRFP–GFP–LC3 reporter (“auto-QC” mouse) emits both green (pH-sensitive) and red (pH-stable) signals: colocalization of GFP and RFP indicates neutral autophagosomes, whereas loss of GFP (with red-only puncta) marks acidified autolysosomes. Using an auto-QC LC3 reporter alongside a complementary mitophagy reporter (“mito-QC”), a recent longitudinal study profiled autophagy in mouse brain across the lifespan. Remarkably, it found that while general macroautophagy puncta in neurons declined modestly with age in select regions (e.g. hippocampal CA1 neurons), mitophagy remained sustained throughout healthy aging in most neurons. This long-term two-photon imaging of live neurons in aging brains (enabled by fluorescent LC3 reporters) underscores how different autophagic pathways (bulk vs. mitochondria-specific) can be uncoupled during aging (Rappe et al., 2024). Such *in vivo* imaging tools now allow researchers to monitor autophagic activity in specific neuronal populations (e.g. dopaminergic neurons, astrocytes, Purkinje cells) under physiological or stress conditions, providing insights into cell-type vulnerabilities and compensatory mechanisms over time.

In cultured neurons, live-cell microscopy with fluorescent LC3/GABARAP reporters has been refined to capture fast autophagic events. Beyond static puncta counts, dynamic assays measure autophagosome formation and clearance rates (flux) using markers like LC3-II turnover or pH-sensitive probes (Kimura et al., 2007; Maday and Holzbaur, 2014). One popular strategy employs the tandem GFP–mCherry–LC3 construct (or newer pHluorin-mCherry variants): GFP fluorescence is quenched upon lysosomal fusion, whereas mCherry persists, so the emergence of red-only puncta quantitatively reports autophagosome-to-autolysosome maturation (Kimura et al., 2007; Zhou et al., 2012). A complementary approach uses LIR-based autophagosome probes: Lee et al. (2017) designed a GFP sensor by fusing an LC3-interacting region (LIR) motif to a short hydrophobic domain (HyD), which binds to endogenous LC3/GABARAP on autophagosomal membranes without perturbing autophagic flux. This LIR-based probe (HyD-LIR-GFP) enables live tracking of autophagosome formation in living cells (including neurons)and can even distinguish LC3A/B-positive vs. GABARAP-positive autophagosome subpopulations by swapping different LIR motifs (Lee et al., 2017).

Additionally, fluorescence resonance energy transfer (FRET)–based biosensors have been developed to visualize specific steps of autophagy. These include an LC3B FRET biosensor that monitors the *in vivo* activity of the cysteine protease autophagy related 4B (ATG4B). In this design, LC3B is flanked by a FRET donor–acceptor pair, and cleavage of the LC3 C-terminus by ATG4B (which primes LC3 for lipidation) produces a measurable change in FRET efficiency. This sensor allows real-time quantification of ATG4B activity and LC3 processing kinetics in neurons exposed to different stimuli, such as nutrient starvation or pharmacological modulators, providing unprecedented temporal resolution (Gokerkucuk et al., 2023). FRET readouts and related biosensors (e.g. Transcription factor E (TFEB) reporters or pH-sensitive lysosomal probes) provide quantitative measurements of autophagy initiation, cargo processing, and lysosomal acidification in living cells. A recent study further developed an Red Green Blue (RGB) triple-fluorophore LC3 sensor that changes color as autophagosomes mature, enabling a single reporter to indicate autophagy stage, with green marking early autophagosomes and red/blue labeling later stages (Kim et al., 2020). Together, these imaging innovations provide powerful visual and quantitative assays to dissect autophagy dynamics, coupling morphology with functional readouts in neurons and glia.

Beyond light microscopy, improved electron microscopy (EM) and correlative imaging have also been applied to LC3 research in brain tissue (Nixon et al., 2005). For example, volume EM and immuno-EM in human and animal models have visualized accumulations of autophagosomes in dystrophic neurites of Alzheimer’s disease brains–reaffirming the blockade of autophagic flux as a pathological feature (massive autophagic vacuole buildup) (Nixon et al., 2005; Treusch et al., 2011). These advanced imaging techniques, combined with AI-driven image analysis, are enabling semi-quantitative mapping of autophagy organelles *in situ*, bridging molecular and ultrastructural data.

### 6.3 Functional and activity-coupled manipulation tools

A frontier in autophagy research is linking autophagic activity with neuronal function and manipulating it in real time. Recent studies show that autophagy can be coupled to neuronal activity states. For instance, presynaptic autophagosome biogenesis increases upon sustained neural firing, suggesting a homeostatic mechanism to eliminate synaptic protein surfeit or damage (Yang et al., 2022). A seminal study in *C. elegans* and mammalian neurons demonstrated that ATG9 vesicles undergo activity-dependent exo-endocytosis at synaptic terminals, effectively linking the synaptic vesicle cycle to autophagosome formation (Yang et al., 2022). Mutations in endocytic proteins (like synaptojanin-1, implicated in familial Parkinsonism) caused ATG9 mislocalization and blunted activity-induced autophagy at synapses. This work highlights how neuronal firing can spatially and temporally trigger autophagic clearance, particularly at synapses prone to protein accumulation. Moving forward, researchers are integrating electrophysiology or Ca^2+^ imaging with autophagy reporters to monitor how bursts of action potentials or synaptic plasticity induction might co-regulate LC3 dynamics in real time (Kuijpers et al., 2021; Kulkarni et al., 2021). Such activity-coupling analyses are critical to parse whether autophagy plays direct roles in synaptic remodeling, neurotransmitter release modulation, or memory consolidation.

On the manipulation front, a suite of optogenetic and chemogenetic tools has emerged to precisely control autophagy signaling pathways in neurons (Figure 4C). An exciting development is the creation of lysosome-targeted optogenetic actuators that enable light-controlled modulation of lysosomal function and autophagic flux (Zeng et al., 2024). Zeng et al. (2024) engineered proton pump and channelrhodopsin variants fused to lysosomal membrane proteins (termed lyso-ArchT, lyso-NpHR, lyso-ChR2). By illuminating cells, researchers could acutely hyperpolarize or depolarize lysosomal membranes and thereby alter luminal pH, enzyme activity, and Ca^2+^ release. Strikingly, blue-light activation of the lysosome-targeted channel rhodopsin lyso-ChR2 triggers strong autophagy by inhibiting mTOR and enhances amyloid-β clearance in neural cell models. This optogenetic lysosome activation also alleviates Aβ-induced pathology in a *C*. *elegans* Alzheimer’s model, improving paralysis phenotypes. This demonstrates the feasibility of optogenetic autophagy induction as a therapeutic strategy (Zeng et al., 2024).

Additionally, optogenetic control has been applied to upstream regulators of autophagy. For example, light-activated systems have been used to recruit pro-autophagy factors such as UNC-51-like kinase 1 (ULK1) or TFEB to defined subcellular compartments, enabling spatially precise activation of the pathway. Optogenetic neuromodulation has also been employed to examine how neuronal network activity shapes autophagy *in vivo*, for instance by photostimulating specific hypothalamic neuron populations and monitoring LC3 (Subramanian et al., 2022). These approaches allow reversible, cell-specific perturbation of autophagy with temporal precision.

Designer Receptors Exclusively Activated by Designer Drugs (DREADDs) are engineered G protein–coupled receptors derived from muscarinic acetylcholine receptors and selectively activated by clozapine-N-oxide (CNO) (Roth, 2016). Activation of hM3Dq engages Gαq–phospholipase Cβ (PLCβ) signaling, resulting in the production of inositol 1,4,5-trisphosphate (IP_3_) and endoplasmic reticulum (ER)–dependent Ca^2+^ release, whereas activation of hM4Di signals through Gαi to inhibit adenylyl cyclase activity and reduce intracellular cyclic adenosine monophosphate (cAMP) levels (Roth, 2016). Notably, these signaling pathways substantially intersect with canonical autophagy-regulatory networks, including Ca^2+^- and cAMP-sensitive nodes that converge on the control of autophagic flux (Bootman et al., 2018; Grisan et al., 2021). Although direct evidence linking DREADD activation to neuronal autophagy regulation remains limited, the close correspondence between chemogenetic signaling cascades and established autophagy-modulating pathways positions DREADDs as a powerful experimental platform for interrogating how neuronal activity-dependent signaling dynamics are translated into autophagic responses.

Cell-type-specific adeno-associated virus (AAV) delivery further enables selective manipulation of autophagy-related pathways in neurons and glial cells through the use of restricted promoters, such as synapsin for neurons and glial fibrillary acidic protein (GFAP) for astrocytes. This approach allows parallel analysis of autophagy across distinct neural cell types within the same tissue context, thereby minimizing confounding environmental variables. By examining neurons and astrocytes under identical experimental conditions, it becomes possible to directly compare basal autophagy levels, stress-induced responses, and differences in cargo selection and handling. As neuronal and glial autophagy are increasingly recognized to play distinct yet interdependent roles in maintaining brain homeostasis, such side-by-side analyses are essential. Nevertheless, studies that directly compare and mechanistically link autophagy regulation across neural cell types remain scarce. Accordingly, cell-type-specific genetic and viral strategies provide a critical framework for elucidating how autophagy is differentially—and coordinately—regulated between neurons and glia in both physiological and disease contexts.

### 6.4 Translational models and clinical perspectives

To bridge bench and bedside, researchers are prioritizing human-based models and biomarkers of autophagy (Figure 4D). Patient-derived iPSC models have become indispensable for studying LC3/GABARAP pathways in a patient-specific context. Neurons and glia differentiated from iPSCs of individuals with autophagy-related genetic disorders or neurodegenerative diseases often recapitulate autophagy defects observed *in vivo*. For example, dopaminergic neurons derived from patients with WDR45 (WIPI4) mutations–which cause β-propeller protein-associated neurodegeneration (BPAN)–show marked autophagy impairment, including reduced LC3-II flux and accumulation of ferritin and other substrates. These patient neurons have significantly shortened axons and increased vulnerability to stress, phenotypes attributed to the inability to clear cargo *via* autophagy (Huang et al., 2025). Treatment of such cells with experimental autophagy activators (e.g. cardiac glycosides or mTOR-independent enhancers) has been reported to restore autophagic flux and improve neuronal survival, offering proof-of-concept for therapy.

Similarly, iPSC-derived neurons from patients with C9ORF72-linked ALS/FTD (an autophagy-related gene) exhibit p62 accumulation and disturbed autophagosome dynamics; parallel iPSC-derived microglia show impaired LC3-associated phagocytosis, contributing to neuroinflammation (Webster et al., 2016). These human cell models allow testing of interventions in a dish, and importantly, they produce translatable readouts (e.g. changes in p62/LC3 levels, clearance of disease proteins) that can guide biomarker development.

Complementing iPSC- and organoid-based platforms, direct reprogramming generates induced neurons/astrocytes from adult somatic cells without a pluripotent intermediate, potentially retaining donor age signatures and enabling matched neuron–astrocyte co-cultures (Mertens et al., 2015; Tang et al., 2017; Quist et al., 2022). Given variability, heterogeneity, limited expandability, and conversion-associated stress, results should be benchmarked against iPSC-derived cells, *postmortem* tissue, and *in vivo* data.

Post-mortem brain analyses remain a cornerstone for validating autophagy changes in human disease. Immunohistochemical and biochemical studies in human brain tissue confirm that many neurodegenerative diseases feature autophagy alterations (Nixon et al., 2005). For instance, autopsy studies in Alzheimer’s disease consistently find massive accumulations of LC3-positive autophagic vacuoles and p62-positive aggregates in affected neurons, reflecting stalled autophagic clearance. In Parkinson’s disease brains, nigral neurons show decreased levels of certain GABARAP/LC3 isoforms and accumulated substrates (like α-synuclein and polyubiquitinated proteins), suggesting autophagy insufficiency may underlie protein aggregation (Tanji et al., 2011). Intriguingly, a recent transcriptomic study reported that apolipoprotein E4 (APOE4) carriers, who are at higher risk for Alzheimer’s disease, show reduced mRNA levels of the autophagy markers LC3 and p62 in both brain tissue and astrocytes. This pattern supports the hypothesis that genetic risk factors for Alzheimer’s disease may, at least in part, act by suppressing basal autophagy (Subramanian et al., 2022). Such observations from human tissues provide impetus to design therapies that can reverse these autophagy deficits *in vivo*.

Looking ahead, developing non-invasive biomarkers to monitor autophagy in patients is a key future direction (Zhang et al., 2025). In this vein, attempts are underway to create PET imaging probes that reflect autophagic activity. While no clinical autophagy PET tracer is yet available, conceptually a radioligand could target an autophagy-specific protein or cargo. For example, it has been proposed to develop PET tracers for p62/SQSTM1 or radiolabeled metabolites that accumulate when autophagy is blocked. Many researchers highlighted the need for a PET tracer detecting autophagy substrates as a potential dynamic readout of autophagy in the living brain (Mizushima, 2018). Additionally, peripheral autophagy markers are being explored: levels of LC3-II, p62, or autophagy-regulating microRNAs in CSF and blood have been examined in small patient cohorts, though standardization is needed. Autophagy-specific PET and fluid biomarkers would greatly facilitate clinical trials of autophagy modulators by confirming target engagement in the CNS (Subramanian et al., 2022).

In summary, tools to study LC3/GABARAP in the nervous system now span spatial and proteomic omics maps, live reporters and sensors, and precise optogenetic, genetic, and pharmacological modulators of autophagy. Applied in human-derived systems and linked to neural function readouts, these approaches are poised to drive translation of autophagy biology into therapies. Growing emphasis on human data—from patient iPSC models, *postmortem* studies, early clinical trials, and emerging biomarkers—shows how autophagy can be monitored and safely enhanced in patients. Integrating these cutting-edge methods across levels of analysis, from molecules to behavior, will reveal new roles for LC3/GABARAP-mediated autophagy in brain physiology and support development of novel treatments in neurology and psychiatry.

## 7 Conclusions and perspectives

Decoding the brain’s “ATG8 code” shows that macroautophagy is highly specialized rather than a uniform process. This review highlights three organizing principles of autophagy specialization in the central nervous system. LC3 and GABARAP subfamilies execute a molecular division of labor, with LC3 proteins primarily mediating cargo recognition and phagophore elongation, and GABARAP proteins driving autophagosome maturation, trafficking, and lysosomal fusion through distinct LIR interactions and stage-specific effectors such as PLEKHM1–HOPS. Neurons and astrocytes then deploy these pathways in complementary ways: neurons rely on LC3/GABARAP for long-range proteostasis along axons and dendrites, whereas astrocytes sustain robust autophagy for extracellular aggregate clearance and transcellular removal of neuronal debris, creating a vulnerable neuron–glia partnership. Finally, autophagy is compartmentalized at synapses, where presynaptic LC3 pathways clear aged vesicles and organelles, postsynaptic autophagy shapes AMPA receptor turnover during long-term depression, and GABARAP-dependent trafficking maintains GABA_A receptor surface expression, so that disruption at specific steps leads to distinct circuit and disease phenotypes.

Several fundamental questions now define the field. A major challenge is to resolve how LC3 and GABARAP are dynamically regulated at individual synapses during learning and memory, which will require combining advanced autophagy reporters with *in vivo* imaging during behavior. Another priority is to elucidate the signals that coordinate neuronal and astrocytic autophagy *in vivo*, including potential cytokine or extracellular vesicle pathways, to enable rational design of combination therapies. Human-specific mechanisms also need clarification, as patient induced pluripotent stem cell models suggest more pronounced autophagy defects than rodent systems, underscoring the importance of systematic cross-species comparisons. In parallel, developing non-invasive imaging and fluid biomarkers to monitor autophagy in patients will be crucial for target engagement and personalized treatment in clinical trials.

Recognizing the ATG8 code argues for moving from broad autophagy stimulation toward precision modulation of LC3- *versus* GABARAP-dependent functions in defined cell types and subcellular compartments. Emerging proof-of-concept studies illustrate this strategy, including astrocyte-targeted LC3B enhancement that limits amyloid-β pathology, proposed boosting of neuronal GABARAP activity to resolve autophagosome–lysosome fusion defects, and compartment-restricted “autophagy on demand” using optogenetic lysosome activation. Key translational goals now include validating rodent mechanisms in human-relevant models, building robust autophagy biomarkers for patient stratification, and achieving reliable cell- and synapse-specific delivery in the human brain to harness LC3/GABARAP autophagy for future therapies in neurology and psychiatry.
